# Factors associated with knowledge about family planning and access to sexual and reproductive health services by sexually active immigrant youths in Hillbrow, South Africa: a cross-sectional study

**DOI:** 10.1186/s12978-022-01477-9

**Published:** 2022-07-30

**Authors:** Nkechi Obisie-Nmehielle, Ishmael Kalule-Sabiti, Martin Palamuleni

**Affiliations:** Population Studies and Demography, North-West University, Mafikeng, South Africa

**Keywords:** Family planning, Sexual and Reproductive Health, Immigrants, Youths, Access to SRH services, Knowledge, Cross-sectional study, Hillbrow, South Africa

## Abstract

**Background:**

In South Africa, universal access to health care services, including those relating to sexual and reproductive health (SRH) care, is contained in Section 27 of the Constitution and commits the country to supporting the United Nations 2030 Agenda for the Sustainable Development Goals (SDGs). The objective of this study was to examine the factors associated with knowledge about family planning and access to SRH services among sexually active immigrant youths in Hillbrow, South Africa.

**Method:**

This cross-sectional study was based on data from a household survey conducted in Hillbrow during December 2019. Interviewer-administered questionnaires were used to collect information from immigrant youths (18–34 years old). Data on 437 sexually active respondents was analysed in STATA 14 using univariate, bivariate, logistic, and multinomial regression models. A p-value of < 0.05 was chosen as the level of significance.

**Results:**

About half of the respondents had poor knowledge about family planning; about one-third (35%) of the immigrant youths had no access to SRH services, 42% had some access, and 23% had access. The adjusted logistic regression model showed that being a female (AOR = 3.85, CI: 2.34–6.35, belonging to age group 30–34 years (AOR = 3.88, CI: 2.00–7.53); belonging to the rich wealth index (AOR = 2.55 (1.32–4.93); not having received information about family planning (AOR = 0.17, CI = 0.10–0.29) and not using a contraceptive at the time of the survey (AOR = 0.37, CI: 0.19–0.70) were factors associated with having knowledge about family planning. The adjusted multinomial regression shows that the factors associated with not having access to SRH services were secondary or higher level of education (ARRR = 1.89, 95% CI = 1.06–3.36), belonging to the rich wealth quintile (ARRR = 2.25, 95% CI = 1.00–5.07), being undocumented (ARRR = 0.49, 95% CI = 0.27–0.88), having experienced discrimination in Hillbrow (ARRR = 2.06, 95% CI = 1.15–3.67) and having received information about family planning 6 months prior to the survey (ARRR = 0.49, 95% CI = 0.26–0.90, p-value < 0.05).

**Conclusion:**

To move towards realization of the Constitution of South Africa, achieve the SDGs, and curb associated negative SRH outcomes, there is a need to advocate for the implementation of universal access to SRH services that is inclusive of immigrant youths.

## Plain language summary

Access to SRH services by adolescents and youths has been a serious public health concern globally. In South Africa, universal access to health care including reproductive health care services is contained in Sect. 27 of the Constitution. The country is committed to the 2030 Agenda of the SDGs with the theme “leaving no one behind”. The objective of this study was to examine the factors associated with knowledge about family planning and access to SRH services among sexually active immigrant youths in Hillbrow, South Africa. Interviewer-administered questionnaires were used to collect information on the socio-demographic, migration, and reproductive health characteristics from 439 sexually active immigrant youths in Hillbrow during December 2019. The data were analysed to assess the factors associated with knowledge about family planning and access to SRH services. The findings show that 46% of the immigrant youth had poor knowledge about family planning, while 35% had no access to SRH services, 42% had some access and only 23% had access. The study showed that the factors associated with knowledge about family planning are female respondents, from the rich income group, those in the older age category, those who received information about family planning 6-months prior to the survey and those using a contraceptive at the time of the survey. The factors associated with having no access to SRH services were those respondents having completed secondary education and higher, from the rich income group, undocumented immigrants, those respondents having experienced discrimination in Hillbrow, youths not having received information about family planning prior to the survey, and those not using a contraceptive. It is important to make access to SRH services available to immigrant youths to prevent associated negative SRH outcomes among the youths.

## Introduction

Access to sexual and reproductive health (SRH) services by adolescents and youths has been a serious public health concern globally since the United Nations (UN) coordinated an international Conference in Cairo in 1994 [1]. Subsequently, the Colombo Declaration on Youth [2] and the UN Sustainable Development Goals (SDGs) 2030 Agenda, [3] recommended universal access to SRH services and information for youths with the pledge of “leaving no one behind” [3]. These are core requirements to ensure that young people, especially young women, can make informed choices about when to have children and prevention of sexually transmitted diseases [4]. Despite these global commitments and targets, there is a dearth of literature on the factors associated with access to SRH services in government health facilities by immigrant youths in a high-migrant populated community like Hillbrow. In South Africa, immigrant youths aged 18–34 accounted for 29.4% of all international migrants and 9.2% of all youths from 18 to 34 years in 2019 [5]. Hillbrow is an immigrant-dense urban suburb of Johannesburg, with a total population of approximately 74,000; of which 58% are between 18 and 34 years, and 27% are immigrant youths [6]. Researchers globally have examined factors influencing knowledge of family planning KFP and access to SRH services by immigrant youths [7, 8]), which include gender, age, level of education, wealth index [9] and migration status [10, 11]. Other influencing factors identified by researchers include experiences of discrimination and lack of social support [12, 13]; as well as attitude of health care practitioners [8, 14].

Increased international migration, as well as the increasing number of undocumented migrants, has heightened concerns about immigrants being exposed to health risks. It is claimed that the South African laws, policies, and guidelines “are progressive and comprehensive on contraceptive service provision in the public sector; and promote integrated, rights-based service delivery” [15]. Negative consequences of poor SRH outcomes—such as unplanned and unwanted pregnancies, HIV/AIDS, sexually transmitted infections (STIs), maternal mortality and infertility—these all affect the development indicators of the country. In order to curb the negative SRH outcomes among the entire population, South Africa is committed to the SDGs, especially SDG 3.7 on universal access to SRH services. Access to health facilities for SRH services is considered vital for all youths. Since sexually active youths interact within the same social space, it is important for all youths to have access to SRH services, irrespective of migration status.

Despite the growing population of immigrant youths—both documented and undocumented, in South Africa, specifically in Hillbrow with the highest population of immigrants in Johannesburg, little is known about their knowledge about family planning. Furthermore, there is scanty of evidence about their access to SRH services from government health facilities in their neighbourhood (Hillbrow Community Health Centre and General Clinic, Hillbrow). Based on the Behavioural Model of Health Service Utilization (BMHSU) and the modified model on health service utilization among immigrants, [16, 17], the main objective of this study was to determine the factors associated with KFP and access to SRH services in government health facilities by sexually active immigrant youths in Hillbrow, South Africa.

## Methods

**Fig. 1:**
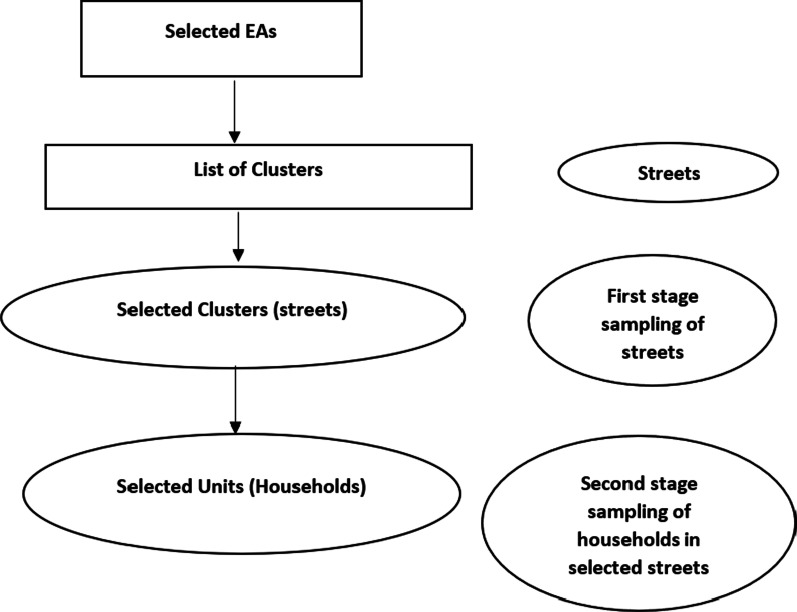
Flow chart of sampling technique

This was a cross-sectional study involving a household survey that used interviewer-administered questionnaires to gather information from immigrant youths (18–34 years), living in Hillbrow, in December 2019. Data on 437 sexually active respondents was analysed in STATA 14 using univariate, bivariate, logistic, and multinomial regression models. A p-value of < 0.05 was chosen as the level of significance. The present study is part of a larger study that examined the “Sexual behaviour of immigrant youths and their access to reproductive health services in Hillbrow, South Africa”. The study population consisted of immigrant youths only, both male and female, 18–34 years of age, who had crossed an international border into South Africa, both documented and undocumented. Data on socio-demographic characteristics, migration, and sexual and reproductive health were collected from consenting immigrant youths. The sample size for frequency in a population with a correction factor was used to calculate the sample size of immigrant youths needed for this study to account for the effect of intra-cluster correlation or the design effect [18, 19]. The Open Epi Version 3.05.07 software for estimating a population proportion with specified absolute precision was used to calculate the sample size. The design effect (DEFF) of 1.25 was used in order to correct for estimated sample variance and account for within group differences among the immigrant youths. The following formula for sample size calculation was used:

1 $$n=\frac{DEFF*Np\left(1-p\right)}{\left[\left(\frac{{d}^{2}}{{z}_{1-\frac{\alpha }{2}}^{2}}\right)*\left(N-1\right)+p*\left(1-p\right)\right]}$$

where, n = sample size, N = Population size of immigrant youth in Hillbrow, p = Probability of exposure in the population, d = level of precision margin of error, deff = design effect = 1.25, Z = the selected critical value of desired confidence level at 1.96 for the 95% CI. Using this equation, 417 samples were required for this study to detect a difference as statistically significant at 5% level of significance assuming a design effect of 1.25. A non-response rate of 12% was factored into this study. This was because of the nature of the primary study that asked questions on sexual behaviours and some undocumented immigrants may be reluctant to participate in the study. This increased the sample size to 467. The design effect of 1.25 was used to correct for estimated sample variance and account for within group differences among the respondents. The study used a two-stage cluster sampling technique due to lack of a sampling frame and cost. The recruitment of subjects involved the selection of nine (9) enumeration areas (EAs) were made of the 30 EAs in Hillbrow. Figure 1 presents a flow chart of the two-stage sampling technique. A list of all the streets in the selected EAs was mapped, to form the sampling frame of the clusters. A sample of the clusters (streets) was selected as the first stage sampling. From each of the selected clusters (streets), a sample of the specified number of units (households) were selected as the second stage sampling, and the households were those included in the survey. One respondent per household was included in the survey. Questionnaires were administered to subjects who met the inclusion criteria for the study. The inclusion criteria for respondents were immigrants, between 18–34 years of age, resident in Hillbrow with the ability to provide informed consent. Exclusion criteria were internal migrants, non-migrants, migrants not residing in Hillbrow, anyone who is mentally ill or drunk. The study followed the assumptions of two-stage clusters sampling of each cluster being internally heterogenous and homogenous to all the other clusters, non-overlapping, and good representation of the study population.

The questionnaire was structured into themes to capture the different characteristics such as socio-demographic, sexual behaviour, knowledge, and information about SRH and migration characteristics of the respondents. Most of the questions were adapted from the survey questionnaire by the African Population and Health Research Centre (APHRC) and the 2016 South African Demographic and Health Survey [20, 21], after an extensive literature review. The questionnaire was pilot tested and revised before the field work was undertaken to collect the data. The outcome variables measured were KFP and access to SRH services from health facility. Table 1a presents the questions and distribution of responses used for generating the outcome variable, KFP. Using principal component analysis (PCA), a composite variable for KFP was generated from two questions on having heard of a family planning method and knowledge of place to obtain contraceptives, each with a list of contraceptive methods. The Cronbach’s alpha for the two variables were 0.83 and 0.86 respectively. The new variables generated were “Heard of contraceptive method” and “Know place to obtain a contraceptive method”. These two variables were used to generate the variable “knowledge of family planning” (KFP) with “inadequate knowledge” or “adequate knowledge” categories. For the second outcome variable, access to health facility for SRH services, respondents were asked about the challenges they have encountered in using the SRH services in the government health facilities in their community. There are two government health facilities in the community—the Community Health Clinic and the municipal clinic, popularly called General clinic. The challenges listed included high cost, discrimination, attitude of health facility staff, waiting time, language barrier, distance, fear and safety concerns, not knowing about services, thinking they do not need services and lack of legal document (immigration permit to be in South Africa). The summary is presented in Table 1b. A composite variable for access to health facility for SRH services was generated using PCA. The new variable—access to health services—has three categories: no access, some access and access. The selection of explanatory variables was based on the conceptual framework for this study, which proposes the influence of demographic factors (age, gender, marital status); socio-economic factors (wealth index, level of education attained); migration and community social factors (migration status, discrimination and xenophobia, social support), and information about SRH (having recently received family planning messages and use of family planning at the time of the study), on access to health facility for SRH services by immigrant youths. The distribution of the outcome variables is shown in Table 1c.

**Table 1:** **a** Knowledge about Family Planning (N = 439). **b** Access to Sexual and Reproductive Health services (N = 439). **c** Knowledge about Family Planning and access to Sexual and Reproductive Health services by immigrant youth

a				
Question	Yes		No	
	Frequency (n)	Percentage (%)	Frequency (n)	Percentage (%)
Question 1: Have you heard of any of the following contraception methods				
Daily pill	368	83.8	71	16.1
Intra uterine device (IUD)	199	45.3	240	54.7
Implants	290	66.1	149	33.9
Male condom	436	99.3	3	0.7
Female condom	399	90.9	40	9.1
Injectables/Depo provera	353	80.6	85	19.4
Emergency contraception	324	74.1	113	25.9
Cycle beads	191	43.5	248	56.5
Vasectomy	144	33.0	293	67.0
Tubal ligation	140	32.0	297	68.0
Question 2: Do you know a place in this area where you can obtain any of the following family planning methods				
Daily pill	353	80.4	86	19.6
IUD	200	45.6	239	54.4
Implants	272	62.0	167	38.0
Male condom	423	96.4	16	3.6
Female condom	390	88.8	49	11.2
Injectables/Depo provera	320	72.9	119	27.1
Emergency contraception	291	66.3	148	33.7
Cycle beads	64	14.6	374	85.4
Vasectomy	118	26.9	321	73.1
Tubal ligation	111	25.3	328	74.7

b				
Question	Yes		No	
	Frequency (n)	Percentage (%)	Frequency (n)	Percentage (%)
Question: Do you have the following challenges in using the SRH services of the health facility in this area?				
High cost	52	11.8	387	88.2
Discrimination	192	43.7	247	56.3
Attitude of the health facility staff	278	63.3	161	36.7
Waiting time	311	70.8	128	29.2
Language barrier	140	31.9	299	68.1
Distance	22	5.0	417	95.0
Safety concerns and fear	100	22.8	339	77.2
I do not know about the services	90	20.5	349	79.5
I do not think I need SRH services	79	18.0	360	82.0
I do not have necessary documentation	156	35.5	283	64.5

c		
Variable	Frequency (n)	Percentage (%)
Knowledge of family planning		
Inadequate knowledge	203	46.2
Adequate knowledge	236	53.8
Access to SRH services		
No access	156	35.5
Some access	182	41.5
Access	101	23.0

Data analysis was conducted using STATA MP 14. Data was analysed at three levels—univariate, bivariate and multivariate analyses. The univariate analysis used descriptive statistics, percentages, and frequencies to describe background characteristics including demographic, socioeconomic, migration and SRH factors of the respondents. The second level of analysis, the bivariate analysis, assessed the associations between the explanatory variables and the two outcome variables using Pearson’s chi-square test. Explanatory variables were considered significant at a p-value of 0.05 or less and 95% confidence interval. The third level of analysis, the multivariate analysis, was conducted to assess the adjusted association of the explanatory variables with KFP and access to SRH services respectively. A multivariate logistic regression model using adjusted odds ratio (AOR) was used to determine the factors associated with KFP in the presence of the explanatory variables. A binary logistic regression model was used because the outcome variable KFP is dichotomous (inadequate knowledge and adequate knowledge), while a multinomial logistic regression model was used for the outcome variable, access to SRH services, which has three categories (no access, some access and access).

## Results

**Fig. 2:**
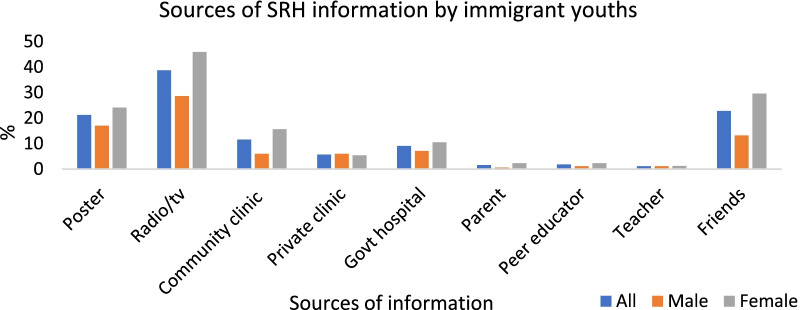
Sources of information about sexual and reproductive health issues by gender

The selected background characteristics of 439 sexually active immigrant youths are summarised in Table 2a, b. About two out of five of the study population were males, while three out of five were females. Those in the age group from 18–24 years constituted two out of five of the study population. Majority, 68% (300) of the immigrant youths have completed secondary education or higher, about 63% (275) were not married, while 40% (176) were in the poor wealth quintile. Over half—54% (239) reported being documented, while 46% (200) reported themselves as undocumented. Over half—58% (254) of the respondents reported having experienced discrimination in Hillbrow, and 79% (346) reported having social support in South Africa. With regards to sexual and reproductive health characteristics, 60% (264) reported having had information about family planning six months prior to the survey. Regarding current use of contraception, 81% (357) reported that they were using a contraceptive method. Figure 2 shows the different sources of information on SRH among sexually active respondents disaggregated by gender. The main sources of information on SRH among all respondents were television and radio—38.7% (170), friends—22.8% (100) and poster 21.2% (93). Community clinic and government hospital were 11.6% (51) and 9.1% (40) respectively. Similarly, among the females, the main sources of information on SRH were television and radio 45.9% (118), friends 29.6% (76) and poster 24.1% (62); while only 15.6% (40) and 10.5% (27) of females sourced information about SRH issues from the community clinic and government hospital respectively. Among the males however, 28.6% (52) got information about family planning from television or radio, while 13% (24) relied on friends. Only a small proportion of males, 6% (11) received information on SRH from the community clinic and 7% (13) from government hospital. Table 1c summarised the distribution of the two outcome variables. For knowledge of family planning, inadequate knowledge and adequate knowledge were 46.2% (203) and 53.8% (236) respectively. For the distribution of access to SRH services, no access, some access and access were 35.5% (156), 41.5% (182) and 23.0% (101) respectively.

**Table 2:** **a** Socio-demographic characteristics of sexually active immigrant youths (N = 439). **b** Reproductive Health characteristics of sexually active immigrant youths (N = 439)

a		
Variable	Frequency (n)	Percentage (%)
Sex		
Male	182	41.5
Female	257	58.5
Age group		
18–24	172	39.2
25–29	126	28.7
30–34	141	32.1
Highest level of education attained		
Primary/incomplete secondary	139	31.7
Complete Secondary/Higher	300	68.3
Marital status		
Single	275	62.6
Married/Cohabiting	164	37.4
Wealth index		
Poor	176	40.1
Middle	163	37.1
Rich	100	22.8

b		
Variable	Frequency (n)	Percentage (%)
Migration status		
Documented	239	54.4
Undocumented	200	45.6
Duration of stay in Hillbrow		
0–2 years	105	23.9
3–5 years	171	39.0
6–20 years	163	37.1
Discrimination in Hillbrow		
Yes	254	57.9
No	185	42.2
Social support in South Africa		
Yes	346	78.8
No	93	21.2
Information about family planning 6 months prior to survey		
Yes	264	60.1
No	175	39.9
Current use of family planning		
Yes	357	81.3
No	82	18.7

### Factors associated with knowledge about family planning among sexually active immigrant youths

The socio-demographic factors that are significantly associated with the knowledge about family planning at bivariate level are presented in Table 3a. The factors were gender (p < 0.001), age group (p < 0.001), highest level of education attained (p < 0.01), marital status (p < 0.01), and wealth index (p < 0.001). The bivariate association between knowledge of family planning, migration and reproductive health characteristics of the immigrant youths is presented in Table 3b. The factors were migration status (p < 0.001), having experienced discrimination in Hillbrow (p < 0.01), having social support in South Africa (p < 0.001), having received information about family planning six months prior to the survey (p < 0.001) and use of family planning at the time of the survey (p < 0.001). Multivariate logistic regression model adjusted for socio-demographic, migration and SRH factors that were significant at bivariate analysis as shown in Table 4. The females were more likely to have knowledge about family planning compared to males (AOR: 3.85, CI = 2.34–6.35). Immigrant youths in the age groups 25–29 years and 30–34 years were more likely to have knowledge of family planning compared to those aged 18–24 years; (AOR:2.14, CI = 1.12—4.05) and (AOR: 3.87, CI = 2.00–7.49) respectively. Belonging to the middle and high wealth indexes increase the likelihood of having knowledge of family planning compared to those in poor wealth index (AOR: 1.83, CI = 1.05—3.18) and (AOR:2.55, CI = 1.32—4.93) respectively. Immigrant youths who did not receive information about family planning 6 months prior to the survey had reduced likelihood of having knowledge about family planning compared with their counterparts that received the information 6 months prior to the survey (AOR: 0.17, CI = 0.10 – 0.29). Similarly, immigrant youths who were not using a family planning method at the time of the survey had reduced likelihood of having knowledge about family planning (AOR: 0.37, CI = 0.19—0.70).

**Table 3:** **a** Association between knowledge about Family Planning and socio-demographic characteristics of sexually active immigrant youths. **b** Association between knowledge about Family Planning and Reproductive Health characteristics of sexually active immigrant youths

a			
Variable	Inadequate knowledge	Adequate knowledge	p-value
	n = 203 (46.2%)	n = 236 (53.8%)	
Sex			< 0.001
Male	122 (67.0)	60 (33.0)	
Female	81 (31.5)	176 (68.5)	
Age group			< 0.001
18–24	99 (57.6)	73 (42.4)	
25–29	55 (43.6)	71 (56.4)	
30–34	49 (34.7)	92 (65.3)	
Highest level of education attained			< 0.01
Primary/Incomplete secondary	78 (56.1)	61 (43.9)	
Complete secondary/Higher	125 (41.7)	175 (58.3)	
Marital status			< 0.01
Single	143 (52.0)	132 (48)	
Married/Cohabiting	60 (36.6)	104 (63.4)	
Wealth index			< 0.001
Poor	107 (60.8)	69 (39.2)	
Middle	65 (39.9)	98 (60.1)	
Rich	31 (31)	69 (69)	

b			
Variable	Inadequate knowledge	Adequate knowledge	p-value
	n = 203 (46.2%)	n = 236 (53.8%)	
Migration status			< 0.001
Documented	86 (36.0)	153 (64.0)	
Undocumented	117 (58.5)	83 (41.5)	
Duration of stay in Hillbrow			0.242
0–2 years	54 (51.4)	51 (48.6)	
3–5 years	71 (41.5)	100 (58.5)	
6–20 years	78 (47.8)	85 (52.2)	
Discrimination in Hillbrow			< 0.01
Yes	105 (41.3)	149 (58.7)	
No	98 (53.0)	87 (47.0)	
Social support in South Africa			< 0.001
Yes	142 (41.0)	204 (59.0)	
No	61 (65.6)	32 (34.4)	
Information about family planning 6 months prior to survey			< 0.001
Yes	70 (26.5)	194 (73.5)	
No	133 (76.0)	42 (24.0)	
Current use of family planning			< 0.001
Yes	151 (42.3)	206 (57.7)	
No	52 (63.4)	30 (36.6)	

***p < 0.001, **p < 0.01, *p < 0.05

**Table 4:** Binary logistic regression of knowledge of Family Planning among sexually active immigrant youths (N = 439)

Variable	Adjusted Odds ratio	95% CI
Sex		
Male	1	1
Female	3.85***	2.34–6.35
Age group		
18–24	1	1
25–29	2.14**	1.12–4.05
30–34	3.87***	2.00–7.49
Highest level of education attained		
Primary/Incomplete secondary	1	1
Complete secondary/Higher	1.22	0.71–2.08
Marital status		
Single	1	1
Married/Cohabiting	1.14	0.66–1.99
Wealth index		
Poor	1	1
Middle	1.83*	1.053–0.18
Rich	2.55**	1.32–4.93
Migration status		
Documented	1	1
Undocumented	0.77	0.46–1.27
Discrimination in Hillbrow		
Yes	1	1
No	1.48	0.87–2.50
Social support in South Africa		
Yes	1	1
No	0.65	0.35–1.20
Information about family planning 6 months prior to survey		
Yes	1	1
No	0.17***	0.10–0.29
Current use of family planning		
Yes	1	1
No	0.37**	0.19–0.70

***p < 0.001, **p < 0.01, *p < 0.05, *AOR* adjusted odds ratio

### Factors associated with access to SRH services from government health facilities by sexually active immigrant youth

The unadjusted multinomial logistic regression for access to SRH services and socio-demographic characteristics is shown in Table 5a. With access to SRH services as the base outcome, the socio-demographic factors associated with no access and some access were highest level of education attained and wealth quintile. The bivariate result showed that immigrant youths with complete secondary education and higher have increased risk to have no access to SRH services in government health facilities (RRR = 2.64, 95% CI = 1.55–4.48, p < 0.001). Similarly, the relative risk for having some access to SRH services for those with secondary education and higher was increased by a factor of 2.55 (RRR = 2.55, 95% CI = 1.53–4.25, p < 0.001). There was an increased relative risk of having no access to SRH services for immigrant youths in rich wealth quintile (RRR = 3.48, 95% CI = 1.67–7.23, p-value < 0.001). Similarly, there was an increased relative risk of having some access by those in the middle (RRR = 2.10, 95% CI = 1.22–3.61, p-value < 0.01) and rich wealth quintiles (RRR = 3.0, 95% CI = 1.42–6.30, p-value < 0.001). Table 5b presented the unadjusted multinomial regression of migration and reproductive health characteristics. With access to SRH services as the base outcome, the migration and reproductive health factors associated with no access were migration status, having received information about family planning 6 months prior to the survey and use of contraceptive methods. The factors associated with having some access were migration status, duration of stay in Hillbrow, having social support in in South Africa, having received information about family planning 6 months prior to the survey and use of contraceptive methods. The relative risk of having no access by undocumented immigrant youths decreased by a factor of 0.37 compared to documented immigrants (RRR = 0.37, 95% CI = 0.22–0.62, p-value < 0.001). Similarly, among those with some access to SRH services, the relative risk of undocumented immigrant youths decreased by a factor of 0.36 (RRR = 0.36, 95% CI = 0.21–0.60, p-value < 0.001). Immigrant youths who have lived in Hillbrow for 3–5 years have increased relative risk for some access to SRH services compared with those with 0–2 years of stay (RRR = 2.35, 95% CI = 1.24–4.45, p-value < 0.01). Similarly, those who have lived for 6–20 years in the community have increased relative risk of having some access (RRR = 1.97, 95% CI = 1.04–3.73, p-value < 0.05). The relative risk of having no access by immigrant youths who did not receive information about family planning 6 months prior to the survey decreased by a factor of 0.48 compared to those who received such information (RRR = 0.48, 95% CI = 0.28–0.80, p-value < 0.01). Similarly, the relative risk of having no access to SRH services by immigrant youths who were not using a contraceptive method at the time of the survey decreased by a factor of 0.56 compared to those who were using contraceptives at the time of the survey (RRR = 0.56, 95% CI = 0.31–0.99, p-value < 0.05).

**Table 5:** **a** Association between access to Sexual and Reproductive Health services and socio-demographic characteristics of immigrant youths. **b** Bivariate association between access to Sexual and Reproductive Health services and Reproductive Health characteristics

a				
Variables	Access to SRH services (N = 439) Base category = Access			
	No access		Some access	
	RRR	CI	RRR	CI
Sex				
Male	1		1	
Female	1.18	0.71–1.96	1.42	0.87–2.33
Age group				
18–24	1		1	
25–29	0.73	0.39–1.38	1.27	0.69–2.32
30–34	0.74	0.41–1.34	0.98	0.55–1.75
Highest level of education attained				
Primary/Incomplete secondary	1		1	
Complete secondary/Higher	2.64***	1.55–4.48	2.55***	1.53–4.25
Marital status				
Single	1		1	
Married/Cohabiting	0.97	0.57–1.64	1.35	0.81–2.23
Wealth index				
Poor	1		1	
Middle	1.14	0.64–2.02	2.10**	1.22–3.61
Rich	3.48***	1.67–7.23	3.00**	1.42–6.30

b				
Variable	Access to SRH services (N = 439) Base category = Access			
	No access		Some access	
	RRR	CI	RRR	CI
Migration status				
Documented	1		1	
Undocumented	0.37***	0.22–0.62	0.36***	0.21–0.60
Duration of stay in Hillbrow				
0–2 years	1		1	
3–5 years	1.20	0.64–2.26	2.35**	1.24–4.45
6–20 years	1.12	0.60–2.00	1.97*	1.04–3.73
Discrimination in Hillbrow				
Yes	1		1	
No	1.39	0.84–2.31	0.63	0.38–1.05
Social support in South Africa				
Yes	1		1	
No	0.72	0.40–1.29	0.53*	0.29–0.95
Information about family planning 6 months prior to survey				
Yes	1		1	
No	0.48**	0.28–0.80	0.29***	0.17–0.48
Current use of family planning				
Yes	1		1	
No	0.56*	0.31–0.99	0.27***	0.14–0.52

***p < 0.001, **p < 0.01, *p < 0.05; *RRR* relative risk ratio, *CI* Confidence interval

The results of adjusted relative risk ratio on factors associated with access to SRH services by immigrant youths is presented in Table 6. With access to SRH services as the base outcome, the factors associated with no access to SRH services were level of education attained, wealth quintile, migration status, having experienced discrimination in Hillbrow and having received information about family planning 6 months prior to the survey. The results showed that immigrant youths with completed secondary education and higher have increased relative risk to have no access to SRH services compared to their counterparts with primary or incomplete secondary education (ARRR = 1.89, 95% CI = 1.06–3.36, p-value < 0.05). Compared with those in the poor wealth quintile, those in the rich quintile had an increased relative risk of having no access to SRH services (ARRR = 2.25, 95% CI = 1.00–5.07, p-value < 0.05). Compared to the documented, undocumented immigrant youths had a reduced risk of having no access to SRH services (ARRR = 0.49, 95% CI = 0.27–0.88, p-value < 0.05). Immigrant youths who reported not having experienced discrimination in Hillbrow were at increased risk for not having access to SRH services (ARRR = 2.06, 95% CI = 1.15–3.67, p-value < 0.05). Compared to immigrant youths who received information about family planning 6 months prior to the survey, those who did not receive such information had reduced risk of no access to SRH services (ARRR = 0.49, 95% CI = 0.26–0.90, p-value < 0.05). Using access as the baseline outcome, the determinants of having some access to SRH services by immigrant youths are being undocumented (ARRR = 0.51, 95% CI = 0.29–0.91, p-value < 0.05), not having received SRH information 6 months prior to the survey (ARRR = 0.38, 95% CI = 0.21–0.70, p-value < 0.01) and not using any family planning methods at the time of the survey (ARRR = 0.31, 95% CI = 0.16–0.62, p-value < 0.001).

**Table 6:** Multinomial logistic regression analysis on access to Sexual and Reproductive Health services by immigrant youths

Variable	Base outcome (Access to SRH services)			
	No access to SRH services (n = 156)		Some access to SRH services (n = 182)	
	ARRR	95% CI	ARRR	95% CI
Sex				
Male	1	1	1	1
Female	0.80	0.44–1.43	0.86	0.49–1.53
Age group				
18–24	1	1	1	1
25–29	0.74	0.36–1.51	1.21	0.55–2.26
30–34	0.75	0.36–1.55	0.72	0.35–1.48
Highest level of education attained				
Primary/Incomplete secondary	1	1	1	1
Complete secondary/Higher	1.89*	1.06–3.36	1.68	0.95–2.95
Marital status				
Single	1	1	1	1
Married/Cohabiting	0.92	0.51–1.74	1.18	0.63–2.20
Wealth index				
Poor	1	1	1	1
Middle	0.94	0.51–1.73	1.59	0.88–2.88
Rich	2.25*	1.0–05.07	1.56	0.68–3.55
Migration status				
Documented	1	1	1	1
Undocumented	0.49*	0.27–0.88	0.51*	0.29–0.91
Discrimination in Hillbrow				
Yes	1	1	1	1
No	2.06**	1.15–3.67	1.02	0.57–1.82
Information about family planning 6 months prior to survey				
Yes	1	1	1	1
No	0.49*	0.26–0.90	0.38**	0.21–0.70
Current use of family planning				
Yes	1	1	1	1
No	0.68	0.36–1.27	0.31***	0.16–0.62

***p < 0.001, **p < 0.01, *p < 0.05; *CI* confidence interval, *ARRR* adjusted relative risk ratio

## Discussion

This study examined the determinants of KFP and access to SRH services provided by government health facilities among sexually active immigrant youths in Hillbrow, South Africa. The study identified inadequate KFP and lack of access and some access to SRH services from government health facilities in Hillbrow as factors contributing to negative SRH outcomes. It further showed that majority of the participating immigrant youth’s sourced information about SRH issues from unreliable sources. They did not get information about SRH from the health facilities in their neighbourhood though they are within walking distance from their residence. This finding is of concern because of the social environment of Hillbrow, which consist of a mix of internal migrants, non-migrants, and international migrants; with lots of social activities including risky sexual behaviours and alcohol consumption [22]. Lack of access to SRH services including correct, reliable information about SRH issues resulted in poor knowledge about family planning and eventually negative SRH outcomes including STIs, re-infections, HIV infections, unplanned and unwanted pregnancies, unsafe abortions, and maternal mortality in Hillbrow, because sexual relationships among youths in Hillbrow do not respect migration status. The observed gender differences, with more males than females lacking knowledge about family planning, may be a consequence of more females seeking SRH information and services due to pregnancy and childbearing needs. Participants in the group with a higher wealth index have better knowledge, but they have no access to government health facilities. This indicates that the rich acquire SRH services, including knowledge about family planning, from sources other than government health facilities. Despite the availability of youth friendly SRH services for adolescents and youth under 25 years of age in Hillbrow, this study shows that immigrant youth aged 18–24 have poor knowledge about family planning. This study further shows that those immigrant youths without documentation and those who have experienced perceived discrimination had no access to SRH services from government health facilities. Qualitative study among the study population will provide a better understanding.

The association between not having received any information about family planning six months prior to the survey with poor knowledge about family planning, having no access or having some access to SRH services underscore the importance of education, information, and communication about family planning among immigrants in Hillbrow. A previous study in South Africa that used a cross-sectional design in migrant-dense areas recorded poor information about SRH services and products among immigrants [20]. Findings of lack of access to SRH services in by immigrants is consistent with a study on pregnant women’s attendance at three urban antenatal care (ANC) settings in Johannesburg, which showed that the Hillbrow health facility had the lowest levels of ANC attendance, HIV testing and the highest HIV prevalence of all three facilities. The study concluded that the low levels recorded could be attributed to perceived or real lack of access to the services by immigrants [23]. The finding that immigrant youths who were not using contraceptives lacked KFP showed the importance of acquiring knowledge preceding use of contraceptive methods. This is consistent with findings by researchers in Sweden that showed that current use of family planning was associated with KFP [14]. Similarly, Bersamin and colleagues [24] found poor knowledge about accessing SRH services among young men. Lack of access to government health facilities left immigrant youth in Hillbrow with no other choice other than to obtain SRH information from unreliable sources. Qualitative studies are necessary to understand the reason for widespread lack of access to government health facilities by immigrant youth in Hillbrow.

The present study should be interpreted with regards to some important limitations. Firstly, it is a cross-sectional study, thus conclusions cannot be drawn on causal relationships. The techniques used cannot predict immigrant youth’s knowledge about family planning and their access to SRH services over a period of time. The selection of the study site was not random, but was purposefully selected based on the prolific number of immigrants in the locality. Additionally, the study did not do a verification of immigration status of the respondents. Some respondents may not have been truthful about their immigration status for fear of being apprehended. This could have resulted in an under-reporting of migration status by undocumented migrants. However, the use of well-trained field workers and gatekeepers that assured respondents that the survey was purely for academic purposes likely mitigated this bias. Recall bias might be a limitation in participants reporting having received information about sexual and reproductive health 6 months prior to the survey. Another limitation is that the outcome variable considered access to SRH services from government health facilities which included the Hillbrow community clinic, and the General clinic (a municipal clinic) did not include the private clinics in Hillbrow. Despite these limitations, the study did however collect quantitative data and has shown the factors associated with knowledge about family planning and access to SRH services by immigrant youths in Hillbrow. The findings of this study could contribute to evidence for migrant-sensitive SRH programming in South Africa.

## Conclusion

Despite the perspective of a progressive approach for rights-based service delivery proposed by South Africa's laws, policies and guidelines on contraceptive service provision in the public sector, this study has shown that in practice the immigrant youths living in Hillbrow lack the necessary access to SRH services in government health facilities. Lack of correct information, knowledge and access to SRH services from health facilities by immigrant youths raises questions about implementation of the policies and guidelines for universal access to SRH services in government health facilities. Considering the high population of youths in Hillbrow and the level of social interactions, including risky sexual behaviours between immigrants and non-immigrants, it is necessary to provide access to SRH services to immigrant youths, in order to prevent re-infections of native-born youths. There is need therefore to advocate for the universal access to SRH services provided by government health facilities to curb the negative SRH outcomes, including unplanned pregnancies, unsafe abortions, HIV infections and re-infections among immigrants and non-immigrant youths, in a social space such as Hillbrow; to enable South Africa to effectively make progress to meet the SDG health targets, especially SDG 3.7 and 5.6, relating to SRH services [3, 4].

## Data Availability

The datasets generated during and/or analysed during the current study are available from the corresponding author on reasonable request.
